# Breaking barriers: noncanonical inflammasome executes blood–brain barrier disruption

**DOI:** 10.1038/s41392-024-01921-1

**Published:** 2024-08-07

**Authors:** Martin Dichgans, Jonas J. Neher, Yaw Asare

**Affiliations:** 1 Institute for Stroke and Dementia Research (ISD), University Hospital, Ludwig-Maximilian-University (LMU), Munich, Germany; 2 Deutsches Zentrum für Neurodegenerative Erkrankungen e. V. (DZNE), Munich, Germany; 3 Munich Cluster for Systems Neurology (SyNergy), Munich, Germany; 4 German Center for Cardiovascular Research (DZHK), Partner Site Munich Heart Alliance (MHA), Munich, Germany; 5 Biomedical Center (BMC), Biochemistry, Faculty of Medicine, LMU Munich, Munich, Germany

**Keywords:** Neuroimmunology, Inflammation

In a landmark study recently published in *Nature*,^1^ Wei and colleagues demonstrate that the activation of GSDMD by the cytosolic lipopolysaccharide (LPS) sensor caspase-11 triggers blood–brain barrier (BBB) breakdown upon LPS challenge independent of TLR4-induced cytokine release. Their work identifies the noncanonical inflammasome and GSDMD pore formation as a potential target for treating central nervous system (CNS) disorders associated with inflammatory BBB dysfunction.

As a specialized interface between the circulatory system and the CNS, the BBB maintains brain homeostasis by regulating the transfer of solutes while limiting the entry of pathogens, toxins, and large molecules.^2^ Pathological states such as infection, trauma, or ischemia can compromise BBB integrity, allowing circulating inflammatory mediators, pathogens, and immune cells to freely enter the brain and trigger neuroinflammation and neuronal injury. Indeed, disruption of the BBB is observed in various neurological disorders, including stroke, Alzheimer’s disease, and multiple sclerosis. LPS, major components of the cell wall of Gram-negative bacteria, elicit a strong pro-inflammatory response and can trigger BBB breakdown, but the mechanisms mediating the loss of barrier integrity are poorly defined. Beyond its recognition at the plasma membrane by Toll-like receptor 4 (TLR4), LPS can be internalized after being bound by CD14 and, in the cytosol, is recognized by inflammatory caspases, which then assemble in a noncanonical inflammasome. This, in turn, leads to cleavage of the protein gasdermin D (GSDMD), which forms a pore in the plasma membrane, triggering pyroptosis.^3^ While this pathway is well-described in macrophages, only recently has data emerged indicating that it can also be activated in non-immune cells.

**Fig. 1:**
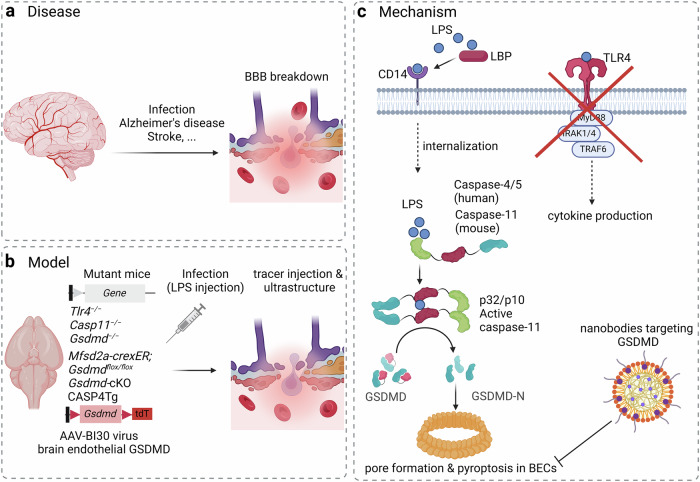
Mechanisms in inflammatory BBB breakdown. **a** Pathological conditions that can compromise BBB integrity. **b** Animal models were used to dissect the mechanisms underlying inflammatory BBB disruption in the study by Wei and colleagues.^1^ Genetic manipulation and adenoviral-mediated transduction of BEC models were challenged with a lethal dose of LPS followed by diverse methodologies to determine the mechanism underlying LPS-induced BBB breakdown. **c** LPS, probably internalized by LBP-CD14 axis, is sensed by the caspase recruitment domain (CARD) of caspase-4/5 (human), or caspase-11 (mouse). This induces the dimerization and activation of inflammatory caspases in a noncanonical inflammasome, causing cleavage of GSDMD to the pore-forming NT-GSDMD fragment that triggers plasma membrane permeability and pyroptosis in BECs. This mode of LPS-induced BBB breakdown is independent of TLR4-mediated cytokine production. Hence, therapeutic strategies for protecting the BBB during bacteremia may involve GSDMD-neutralizing nanobodies. The figure was generated with BioRender.com

In their recent article,^1^ Wei and colleagues now highlight a novel role of this pathway in driving endothelial pyroptosis and BBB disruption in response to high-dose LPS or sepsis. To investigate the mechanism of LPS-induced BBB breakdown, the authors initially injected wild-type, *Tlr4*^*−/−*^, *Casp11*^*−/−*^, and *Gsdmd*^*−/−*^ mice with a lethal dose of LPS. While the LPS challenge led to extensive BBB leakage in wild-type mice, animals deficient in *Tlr4*, *Casp11*, or *Gsdmd* were largely protected against BBB breakdown. Interestingly, the most common location of BBB disruption was in large (diameter > 10.5 µm) arteries and veins with little disruption at the capillary level, demonstrating vascular zonation-dependent effects. They next investigated the LPS-TLR4 axis in this mechanism by challenging mice with two cardinal cytokines released downstream of TLR4 activation, TNF or IL-6. Surprisingly, neither TNF nor IL-6 triggered BBB breakdown, suggesting the involvement of an alternative signaling pathway. Hence, the authors scrutinized the role of the noncanonical inflammasome (Fig. 1). Specifically, they used the TLR3 agonist Poly (I:C) to induce the expression of *Casp11*, which rendered *Tlr4*^*−/−*^ mice, but not *Casp11*^*−/−*^, or *Gsdmd*^*−/−*^ mice, susceptible to LPS-induced BBB disruption. This mode of BBB breakdown requires internalization of circulating LPS, probably through the LBP-CD14 axis, to activate caspase-11. Together, these findings implicated the caspase-11-GSDMD axis in LPS-induced BBB breakdown, independent of TLR4-mediated cytokine production.

The BBB is composed of brain endothelial cells (BECs) interconnected by tight junctions and reinforced by mural cells and astrocytes, all of which are crucial for a functional BBB. To identify the cell types mediating LPS-induced BBB breakdown, the authors profiled *Gsdmd* expression and found the highest levels in BECs and microglia/macrophages. Prioritization of cell types responding to challenges with LPS identified BECs as the most prominent and probably first-line responders to the inflammatory assault. Furthermore, *Casp11* and *Cd14* expression increased in BECs post-LPS, suggesting their involvement in the LPS-induced BBB leakage. Functional assays in mouse primary BECs confirmed LPS-induced GSDMD cleavage, plasma membrane permeabilization, and pyroptotic cell death, suggesting their direct sensing of LPS through caspase-11-mediated GSDMD activation.

Having identified a major pathway that mediates inflammatory BBB breakdown and the main cell type involved, the authors next determined the ultrastructural characteristics of LPS-induced BBB disruption. Using an optimized electron microscopy protocol to compare brain areas with and without BBB breakdown, they found pronounced structural abnormalities in BEC post-LPS challenge featuring early pyroptotic morphologies, including condensed nuclei, shrunken cell bodies, and abnormal tight junctions in wild-type but not in *Gsdmd*^*−/−*^ mice. As an additional step, they used cell type-specific genetic manipulation and adenoviral-mediated transduction of BECs to show that *Gsdmd* expression in BECs is indeed required for LPS-induced BBB disruption. Conversely, reintroducing GSDMD exclusively into BECs of *Gsdmd*-deficient mice restored BBB disruption following LPS exposure. Collectively, these data demonstrated the pivotal role of endothelial GSDMD in mediating LPS-induced BBB disruption.

As shown by a range of studies, the mechanism and consequence of GSDMD cleavage may vary depending on cell type and can be independent of inflammatory caspases. In neutrophils, the activation of GSDMD is facilitated by cathepsin G and elastase, contributing to the formation of neutrophil extracellular traps. In macrophages, GSDMD can be activated by caspase-8, in addition to caspase-1 and 11, resulting in a pore-forming NT-GSDMD fragment that triggers pyroptosis. In contrast, cleavage by caspase-3 or caspase-7 occurs at a distinct site in NT-GSDMD (Asp87 in humans, Asp88 in mice), abolishing its pyroptotic ability.^4^ Hence, Wei and colleagues set out to prove that upon LPS challenge, GSDMD would cause the formation of a pore in BECs that allows for bidirectional passage of solutes through these cells. To this end, they generated mouse models with robust tdTomato expression, specifically in BECs that were either sufficient or deficient in *Gsdmd*. Strikingly, after the LPS challenge, *Gsdmd*^*+/+*^ BECs did not only show leakage of tdTomato but also the uptake of the tracer used to detect BBB breakdown (sulfo-NHS–biotin) while these effects were absent in *Gsdmd*^*−/−*^ BECs. These results demonstrate that GSDMD pores allow free passage of solutes through BECs after the LPS challenge. As a final experiment to unequivocally establish the role of GSDMD in BBB breakdown, the authors also demonstrated that direct activation of brain endothelial GSDMD could induce BBB leakage independently of LPS, defining GSDMD as a crucial mediator of BBB integrity.

Importantly, human endothelial cells constitutively express caspase-4, which shows higher LPS sensitivity than caspase-11, rendering humans more sensitive to LPS toxicity.^5^ Therefore, to assess the clinical relevance of this newly identified mechanism mediating BBB disruption, the authors studied mice with human caspase-4 expression (CASP4Tg). Indeed, in CASP4Tg animals, even low-dose LPS was sufficient to disrupt the BBB in a GSDMD-dependent manner, demonstrating the translational relevance of the data. Moreover, therapeutic inhibition of GSDMD using a blocking nanobody (VHH_GSDMD-1_) effectively prevented LPS-induced BBB breakdown, suggesting a potential strategy for BBB protection during infection.

In summary, the study by Wei et al. not only defines a critical pathway that mediates LPS-related BBB leakage but also introduces a novel mechanism of BBB breakdown characterized by plasma membrane permeabilization by GSDMD pores. Traditionally, BBB breakdown has been related to an impairment of endothelial tight junctions, an increase in endothelial transcytosis, or a shift in transport from ligand-specific receptor-mediated to non-specific caveolar transcytosis, as is also seen with physiological aging. Whether the plasma membrane permeabilization by GSDMD pores can cause BBB leakage independent of BEC lysis requires further investigation, as does the question of whether the effects of plasma membrane permeabilization on BBB breakdown are partly mediated by indirect effects on tight junctions and transcytosis. Nevertheless, the study by Wei and colleagues undoubtedly represents a major step forward and offers new molecular targets for therapies aimed at preserving BBB integrity.
